# Molecular characterization of an anthocyanin-related glutathione *S*-transferase gene in Japanese gentian with the CRISPR/Cas9 system

**DOI:** 10.1186/s12870-020-02565-3

**Published:** 2020-08-06

**Authors:** Keisuke Tasaki, Momo Yoshida, Minori Nakajima, Atsumi Higuchi, Aiko Watanabe, Masahiro Nishihara

**Affiliations:** 1 Iwate Biotechnology Research Center, 22-174-4 Narita, Kitakami, Iwate 024-0003 Japan; 2 Present address: Tokyo University of Agriculture, 1737 Funako, Atsugi, Kanagawa 243-0034 Japan; 3 Tokyo University of Agriculture, 1737 Funako, Atsugi, Kanagawa 243-0034 Japan

**Keywords:** Anthocyanin, CRISPR/Cas9, Flower pigmentation, Genome editing, Gentian, GST

## Abstract

**Background:**

The blue pigmentation of Japanese gentian flowers is due to a polyacylated anthocyanin, gentiodelphin, and all associated biosynthesis genes and several regulatory genes have been cloned and characterized. However, the final step involving the accumulation of anthocyanins in petal vacuoles remains unclear. We cloned and analyzed the glutathione *S*-transferases (GSTs) in Japanese gentian that are known to be involved in anthocyanin transport in other plant species.

**Results:**

We cloned *GST1*, which is expressed in gentian flower petals. Additionally, this gene belongs to the Phi-type GST clade related to anthocyanin biosynthesis. We used the CRISPR/Cas9-mediated genome editing system to generate loss-of-function *GST1* alleles. The edited alleles were confirmed by Sanger and next-generation sequencing analyses. The *GST1* genome-edited lines exhibited two types of mutant flower phenotypes, severe (almost white) and mild (pale blue). The phenotypes were associated with decreased anthocyanin accumulation in flower petals. In the *GST1* genome-edited lines, sugar-induced stress conditions inhibited the accumulation of anthocyanins in stems and leaves, suggestvhing that *GST1* is necessary for stress-related anthocyanin accumulation in organs other than flowers. These observations clearly demonstrate that *GST1* is the gene responsible for anthocyanin transport in Japanese gentian, and is necessary for the accumulation of gentiodelphin in flowers.

**Conclusions:**

In this study, an anthocyanin-related *GST* gene in Japanese gentian was functionally characterized. Unlike other biosynthesis genes, the functions of *GST* genes are difficult to examine in in vitro studies. Thus, the genome-editing strategy described herein may be useful for in vivo investigations of the roles of transport-related genes in gentian plants.

## Background

The blue flowers of cultivated Japanese gentians are due to the accumulation of a polyacylated anthocyanin, gentiodelphin [delphinidin 3-*O*-*β*-D-glucosyl-5-*O*-(6-O-caffeoyl-*β*-D-glucoside)-3′-*O*-(6-*O*-caffeoyl-*β*-D-glucoside)], which is a major pigment [1]. Additionally, intramolecular copigmentation is responsible for this blue coloration and pigment stabilization [2, 3]. To clarify the molecular mechanism mediating the development of blue gentian flowers, the genes involved in the associated metabolic pathway have been isolated and characterized. These include biosynthesis genes encoding chalcone synthase (*CHS*), chalcone isomerase (*CHI*), dihydroflavonol 4-reductase (*DFR*), flavanone 3-hydroxylase (*F3H*), flavonoid 3′-hydroxylase (*F3’H*), flavonoid 3′,5′-hydroxylase (*F3’5’H*), flavone synthase II (*FNSII)*, and anthocyanidin synthase (*ANS*), as well as modification genes, such as those encoding anthocyanin 5-glucosyltransferase (*5GT*), 3′-glucosyltransferase (*3′GT*), and 5,3′-aromatic acyltransferase (*5/3’AT*), and regulatory genes encoding MYB and bHLH transcription factors. The transcriptional regulation of flower petal pigmentation has been analyzed in previous investigations involving natural mutant gentian cultivars/lines [4], the overexpression of a chimeric repressor (e.g., SRDX-GtMYB3) via stable transformation [5], and a viral vector [6]. The transcriptional regulation of gentians determined with these approaches have been reviewed in detail [7]. The information was also applied to develop PCR-based molecular markers for distinguishing plants that produce white or pink flowers from those that produce blue flowers [8, 9]. The early selection of progeny based on these molecular markers may be useful for promoting Japanese gentian breeding [10]. However, to the best of our knowledge, the gentian genes related to anthocyanin transport have not been characterized.

In higher plants, anthocyanins biosynthesized in the cytoplasm are transferred to vacuoles via several pathways related to transporters such as the ATP binding cassette (ABC) and glutathione *S*-transferase (GST). Pioneering studies revealed that maize *Bronze2* (*Bz2*) and petunia *Anthocyanin9* (*An9*), which encode GSTs, are responsible for the vacuolar accumulation of anthocyanins [11]. Orthologous *GST* genes related to anthocyanin accumulation have been identified in several ornamental flowering plants, such as *DcGSTF2* in carnation [12] and *CkmGST3* in cyclamen [13], but in relatively few other species. Although the genes encoding putative GST and ABC proteins responsible for anthocyanin transport have been detected in many plant species and are available in public databases, only a few have been functionally characterized. One of the reasons for this is that analyzing anthocyanin transport is difficult in vivo. Unless mutant lines are available, confirming gene functions is difficult and the transport of anthocyanin pigments to vacuoles has been examined mainly in in vitro uptake assays with artificial membrane vesicles. Some studies involving the genetic complementation of the Arabidopsis *tt19* mutant line revealed potential functions of GSTs [13–16], but it remains unclear whether the GSTs are indeed functional in the original plant species. Virus-induced gene silencing is also useful for analyzing gene functions, but it is often difficult to apply this system to horticultural crops and a family of highly homologous genes.

The clustered regularly interspaced short palindromic repeats (CRISPR)/Cas9 system is the most promising genome editing tool in higher plants [17, 18], and novel CRISPR technologies are expected to make important contributions to plant science research [19]. The CRISPR/Cas9 system has been used to investigate several ornamental flower species by targeting specific genes such as *DFR* in Japanese morning glory (*Ipomoea nil*) [20] and *F3H* in torenia [21]. Additionally, we were the first to establish a CRISPR/Cas9 system targeting phytoene dehydrogenase (*PDS*) to examine gentians [10]. Moreover, we completed an in vivo functional analysis of anthocyanin modification genes, including *5GT*, *3’GT*, and *5/3’AT* [22]. Thus, the CRISPR/Cas9 system can now be used to accelerate functional analyses of genes in gentians, which are non-model plants.

The objective of this study was to identify an anthocyanin-related *GST* gene in Japanese gentians and characterize its function via genome editing with a CRISPR/Cas9 system. A gentian *GST* gene (*GST1*) was cloned and confirmed as responsible for anthocyanin accumulation. We demonstrated that this gene influences the accumulation of anthocyanins in both gentian flowers and leaves. To the best of our knowledge, this is the first report of the application of a CRISPR/Cas9 system to study anthocyanin transport-related genes in higher plants. Furthermore, our findings confirm that the genome-editing strategy applied in this study is useful for identifying uncharacterized gentian genes.

## Results

### Isolation and identification of a *GST* gene related to anthocyanin pigmentation

**Fig. 1:**
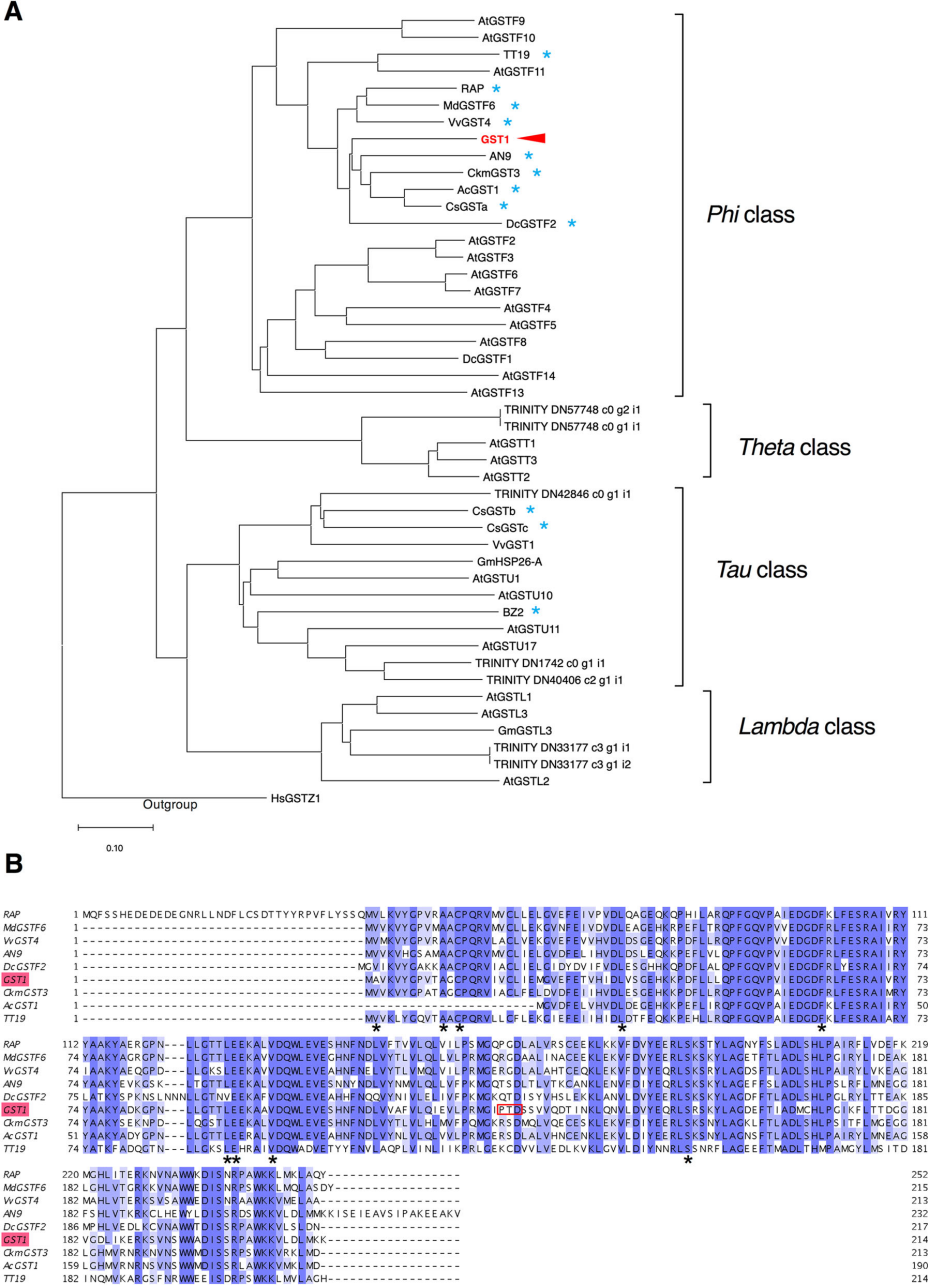
Phylogenetic tree and alignment of GSTs. **a** A phylogenetic tree comprising GSTs was constructed based on amino acid sequences according to the neighbor-joining method (1000 bootstrap replicates) with the CLUSTALW (Thompson et al. 1994) and MEGA programs. Blue asterisks indicate anthocyanin-related GSTs. **b** Alignment of anthocyanin-related GSTs with CLUSTALW and Jalview (version 2.10.3). Asterisks indicate the positions of conserved amino acid residues specific to anthocyanin-related GSTs. The red box indicates the three deleted amino acid residues due to the deletion in allele 1 of *GST1* genome-edited line #3. The accession numbers of GSTs used for constructing the phylogenetic tree and the sequence alignment are listed in Additional file 1: Table S4

Full-length sequences of two alleles of a deduced *GST* gene were determined by sequencing the PCR products amplified from cDNA samples derived from gentian cultivar ‘Albireo’ petals. The coding region sequences of the deduced *GST* were 642 bp long, with three single nucleotide polymorphisms (SNPs) between the two alleles, which both encode proteins consisting of 214 amino acid residues, with one amino acid difference at position 114 (GST1–1, phenylalanine; GST1–2, leucine). We named these alleles *GST1–1* (accession no. LC536038) and *GST1–2* (accession no. LC536039), and hereafter refer to them together as *GST1*. The raw sequencing data are available in the DDBJ Sequence Read Archive (DRA) database (accession no. DRA010021). An analysis of the *GST1–1* and *GST1–2* genome sequences (accession nos. LC536040 and LC536041) revealed these two alleles comprise 1623 and 1667 bases from the start to stop codons, respectively. A phylogenetic analysis indicated that GST1 belongs to the Phi-type GST group (Fig. 1a). The sequence identity between the deduced GST1 and Phi-type GSTs related to anthocyanin biosynthesis ranged from 53 to 66%: RAP (66%), VvGST4 (66%), AN9 (65%), DcGSTF2 (62%), MdGSTF6 (66%), CkmGST3 (68%), and TT19 (53%). Additionally, conserved amino acid residues specific to anthocyanin-related GSTs were detected in GST1 (Fig. 1b).

### Production of *GST1* genome-edited gentian lines

**Fig. 2:**
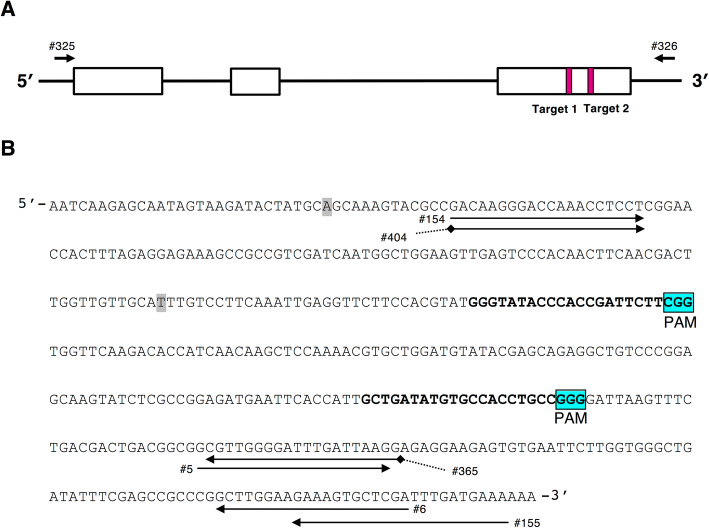
Schematic diagram of *GtGST1*. **a** Schematic diagram of the *GST1* genomic structure. White boxes indicate the exons. Arrows indicate the primer positions. Details regarding the primers are listed in Additional file 1: Table S3. Shaded red boxes represent target sites 1 and 2 for the genome editing with the CRISPR/Cas9 system. **b** Third exon sequence containing target sites 1 and 2 for genome editing. Shaded single nucleotides are the SNPs between two alleles. Bold nucleotides represent the sequences of target sites 1 and 2, and the adjacent blue shaded boxes (PAM) indicate the protospacer adjacent motif (NGG). Arrows indicate the primer positions

The *GST1* genomic sequence includes three exons and two introns. We targeted two sites on the third exon of *GST1* (Fig. 2a) for genome editing with the CRISPR/Cas9 system. By applying *Agrobacterium tumefaciens*-mediated transformation, we obtained 54 bialaphos-resistant transgenic plants and confirmed they were correctly transformed by the genomic PCR amplification of a partial *Cas9* sequence. These lines were prescreened by analyzing the sequences of PCR amplicons including target sites. Consequently, eight lines were selected, and after subcloning, Sanger sequence analyses were performed. The results indicated that seven lines were genome-edited lines in which the targeted *GST1* sites were mutated (Additional file 1: Table S1). All seven of the *GST1* genome-edited lines were acclimated and grown in a closed greenhouse until flowering.

### Next-generation sequencing (NGS) analysis of *GST1* genome-edited gentian plants

The plants bloomed at approximately 4 months after they were first acclimated. Among the seven *GST1* genome-edited lines, changes to the flower color phenotypes were observed in five lines (#3, #12, #13, #23, and #29). Line #1 produced flowers that were similar to wild-type (WT) flowers regarding color and line #8 did not bloom. Therefore, the amplicon sequences of the target sites in the petal transcripts and genomic DNA of these five lines were analyzed with an NGS platform. Amplicon fragment sequences were merged based on the duplicating sequence region between high-quality filtered paired-end reads. We obtained fragments constructed from 70.5–99.9% of the read pairs in each library derived from the transcripts and genomic DNA of WT and *GST1* genome-edited lines. After non-specific or low-copy fragments thought to be derived from PCR and/or sequencing errors were removed, the major fragments of each allele were counted. The proportion of major fragments in each library ranged from 86.9 to 90.2%. The two alleles were distinguished based on the SNPs in the sequenced amplicon region (Fig. 2b). The sequencing results for target sites 1 and 2 in genomic DNA and the transcripts of *GST1* genome-edited lines #3 and #12 are summarized in Table 1, with data for the other lines presented in Additional file 1: Table S2 and Additional file 3. The number of DNA fragments between alleles was almost equal and/or less than 2-fold different. Regarding the transcript analysis, there were more allele 1 fragments than allele 2 fragments in all plants. In lines #3 and #29, no DNA and transcript fragments corresponding to allele 1 were detected. The mutations almost corresponded with the results of the Sanger sequencing analyses presented in Additional file 1: Table S1. Amplicon sequencing analyses of the *GST1* genome-edited lines uncovered several characteristic mutations, such as the deletions of 2, 7, 9, and 19 bases in target sites 1 and/or 2, or more than 100 bases between target sites 1 and 2 (Table 1, Additional file 1: Table S2). Lines #12, #13, and #23 were confirmed to have heterozygous biallelic mutations. In line #3, nine nucleotides in target site 1 were deleted, likely resulting in the deletion of three amino acids in the encoded protein. We detected a consistent mutation pattern between the genomic DNA and transcripts in all *GST1* genome-edited lines.

**Table 1:**
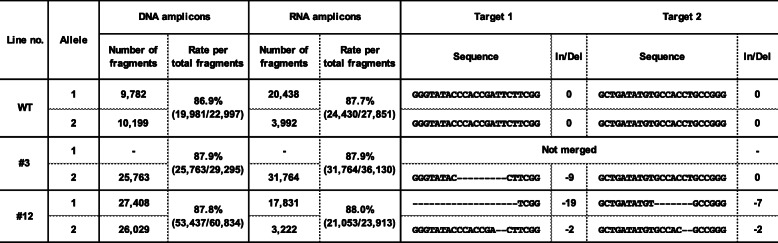
NGS analysis of PCR amplicons of DNA and RNA of *GST1* genome-edited lines

### Petal phenotypes of genome-edited gentian plants

**Fig. 3:**
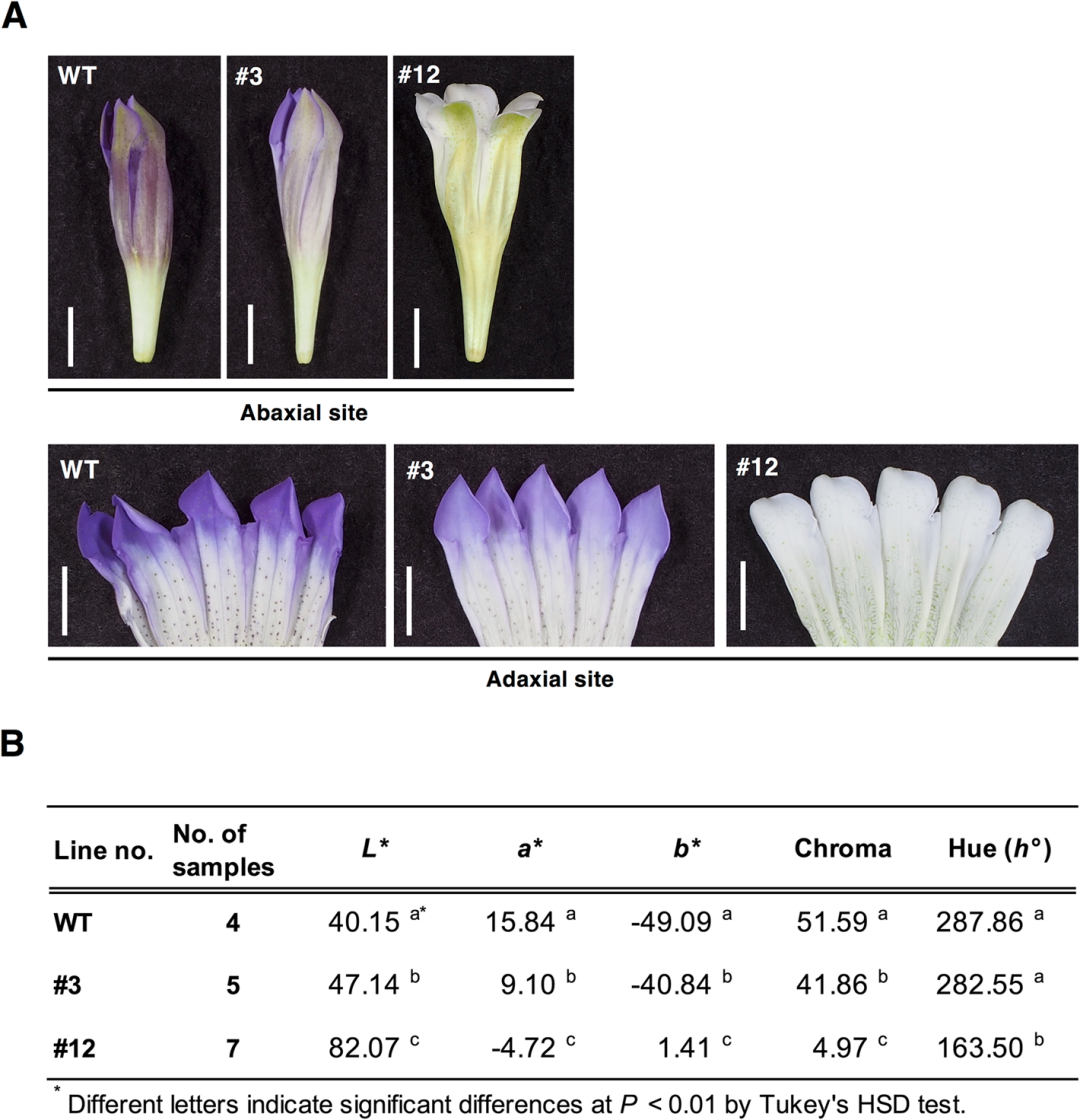
Flower color characteristics in *GST1* genome-edited gentian lines. **a** Flower phenotypes of the wild-type control and *GST1* genome-edited lines #3 and #12. Bar = 1 cm. **b**L*, a*, and b* color values at the surface of fresh petals were measured with the CM-3600A spectrophotometer (Konica Minolta, Tokyo, Japan). The chroma values and hue angles were also calculated

Typical flowers of *GST1* genome-edited lines are presented in Fig. 3a and Additional file 2: Figure S1A. The flowers of lines #12, #13, #23, and #29 were almost completely white. In contrast, the line #3 flowers were similar to the WT flowers, but they were paler. The colorimetric values of the adaxial side of the flower limb area for *GST1* genome-edited lines #3 and #12 are summarized in Fig. 3b. The colorimetric traits of the adaxial surface of petals were analyzed with a spectrophotometer and evaluated based on CIE L*a*b*. To describe the color tone, the hue angle and chroma were calculated based on the recorded data (a* and b*). The L*, a*, b*, chroma, and hue angle values of line #12 were significantly higher and/or lower than those of the WT plants. The L*, a*, b*, and chroma values of line #3 were also significantly different from those of the WT plants, but there was no significant difference in the hue angle. These results confirmed the visible differences between the WT control and *GST1* genome-edited lines. The corresponding values of the other lines are summarized in Additional file 2: Figure S1B. The colorimetric values of lines #13, #23, and #29 were similar to those of line #12, but differed from those of line #3.

### Analysis of anthocyanin compositions in genome-edited gentian plants

**Fig. 4:**
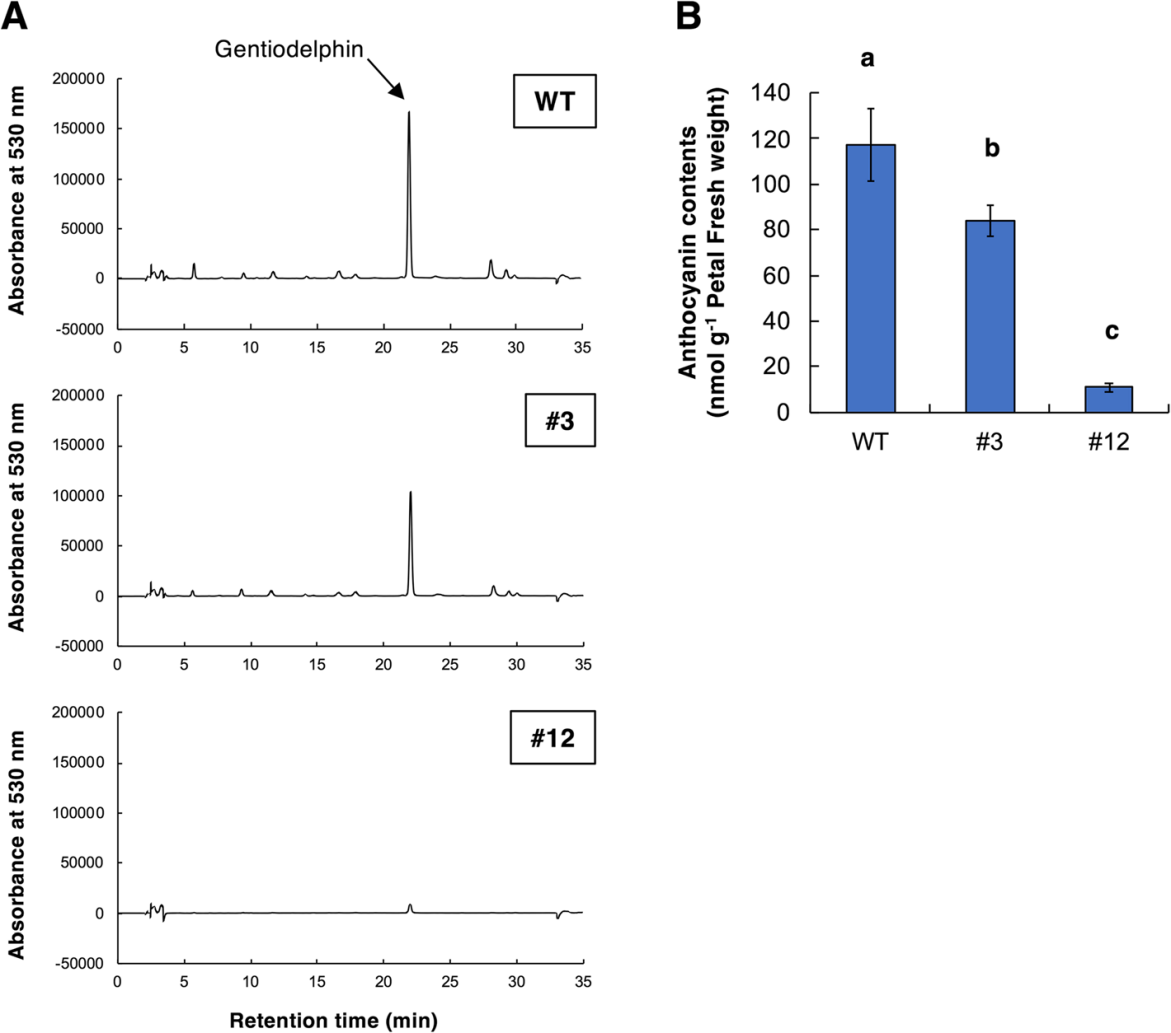
Analysis of anthocyanin accumulation in the petals of *GST1* genome-edited gentian lines. **a** Chromatogram at 530 nm for the anthocyanins in petal extracts from WT plants and *GST1* genome-edited lines. **b** Total anthocyanin contents in the flower petals of *GST1* genome-edited lines #3 and #12 and the WT control. Quantities were estimated based on the molar absorptivity of delphinidin [ε mol = 506,783 at 530 nm, evaluated in an 80% methanol solution containing 0.1% (v/v) trifluoroacetic acid]. Error bars represent the standard error of the means of four petals. Different letters indicate statistically significant differences according to Tukey’s HSD test (*P* < 0.01)

To investigate whether the accumulated pigments in the petals of *GST1* genome-edited lines changed, anthocyanin extracts were analyzed by HPLC at a monitoring wavelength of 530 nm. The chromatograms of *GST1* genome-edited lines #3 and #12 and the WT control are presented in Fig. 4a. The chromatogram of the WT plants included a single peak representing gentiodelphin, whereas the chromatograms of *GST1* genome-edited lines #12 and #3 had an extremely small (ca. 6.1% of the WT) and an intermediate-sized (ca. 63.4% of the WT) corresponding peak, respectively. Additionally, there were no obvious peaks that were detected for lines #3 or #12 that were absent from the WT chromatogram. The total anthocyanin contents in the flower petals of the genome-edited and WT gentian plants were estimated by spectrophotometry (Fig. 4b). Consistent with the HPLC analysis, the total anthocyanin contents were lower in the *GST1* genome-edited lines than in the WT control, with the anthocyanin levels in lines #3 and #12 approximately 71 and < 10% of that of the WT plants, respectively.

### Expression analysis of flavonoid biosynthesis-related genes

**Fig. 5:**
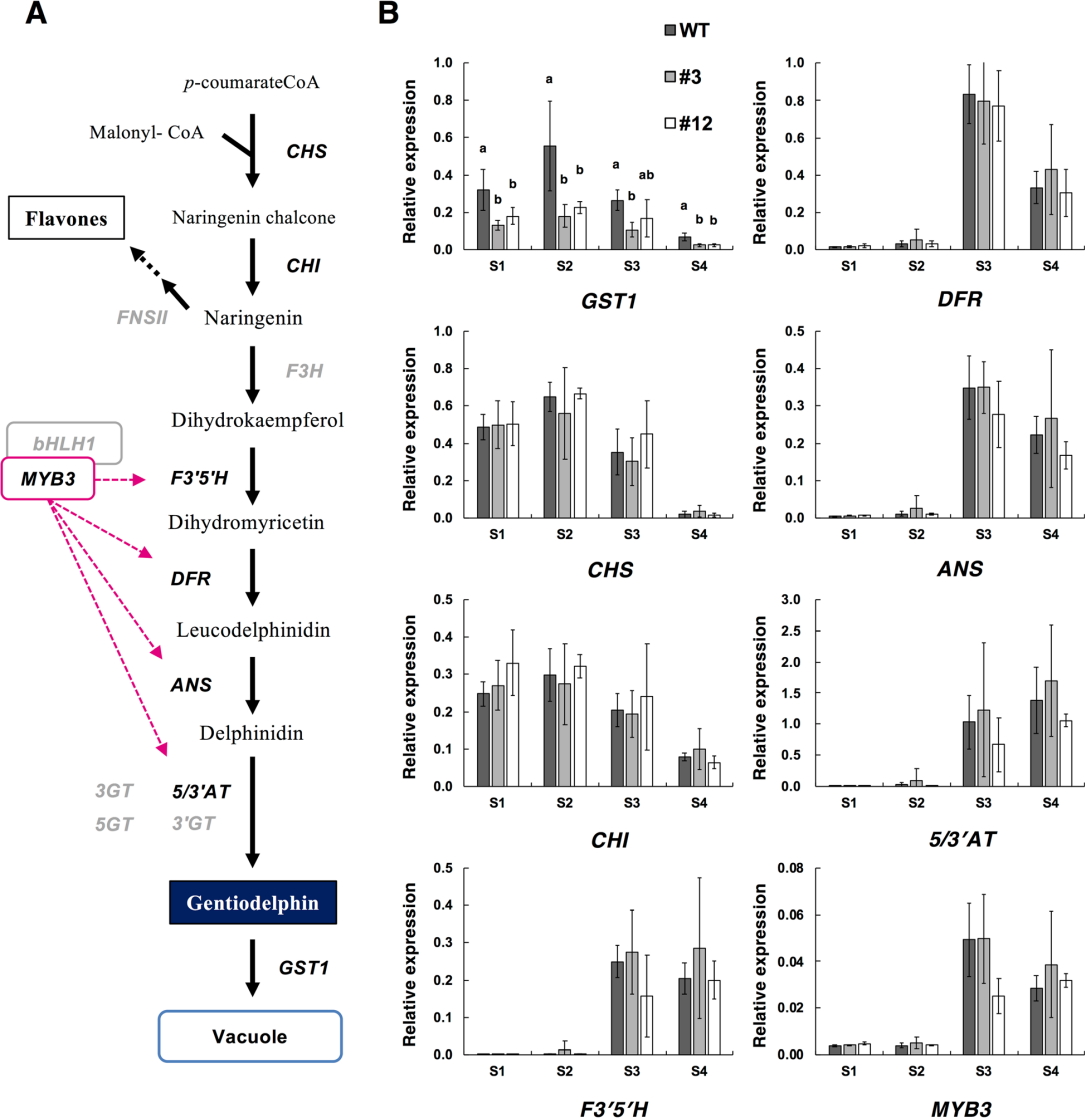
Expression patterns of *GST1* and flavonoid biosynthesis-related genes. **a** Schematic diagram of the flavonoid biosynthesis pathway. Target genes analyzed by qRT-PCR are indicated with bold black uppercase letters. **b** Expression pattern based on the qRT-PCR analysis of petals at floral development stages 1–4. Error bars represent the standard deviation of the means of five samples from individual plants. Different letters indicate statistically significant differences according to Tukey’s HSD test (*P* < 0.05)

Quantitative reverse transcription PCR (qRT-PCR) assays were completed to investigate the expression of *GST1* and flavonoid biosynthesis-related genes during petal development. We selected two biosynthesis genes (*CHS* and *CHI*) as the early genes and four biosynthesis genes (*F3’5’H*, *DFR*, *ANS*, and *5/3′AT*) as the late genes. The *MYB3* transcription factor gene, which regulates the expression of the late biosynthesis genes, was also included in the expression analysis. The resulting data for four developmental stages (S1–S4) (Fig. 5) revealed that the expression pattern of *GST1* in WT plants was similar to those of the early genes, *CHS* and *CHI*. Moreover, the *GST1* expression levels were significantly lower in *GST1* genome-edited lines #3 and #12 (ca. 32.2–63.5% of the WT) than in the WT control. There were no major differences in the expression levels and patterns of the early genes (*CHS* and *CHI*), late genes (*F3’5’H*, *DFR*, *ANS*, and *5/3′AT*), and *MYB3* between the *GST1* genome-edited lines and the WT plants.

### Complementation of the edited *GST1* by particle bombardment

**Fig. 6:**
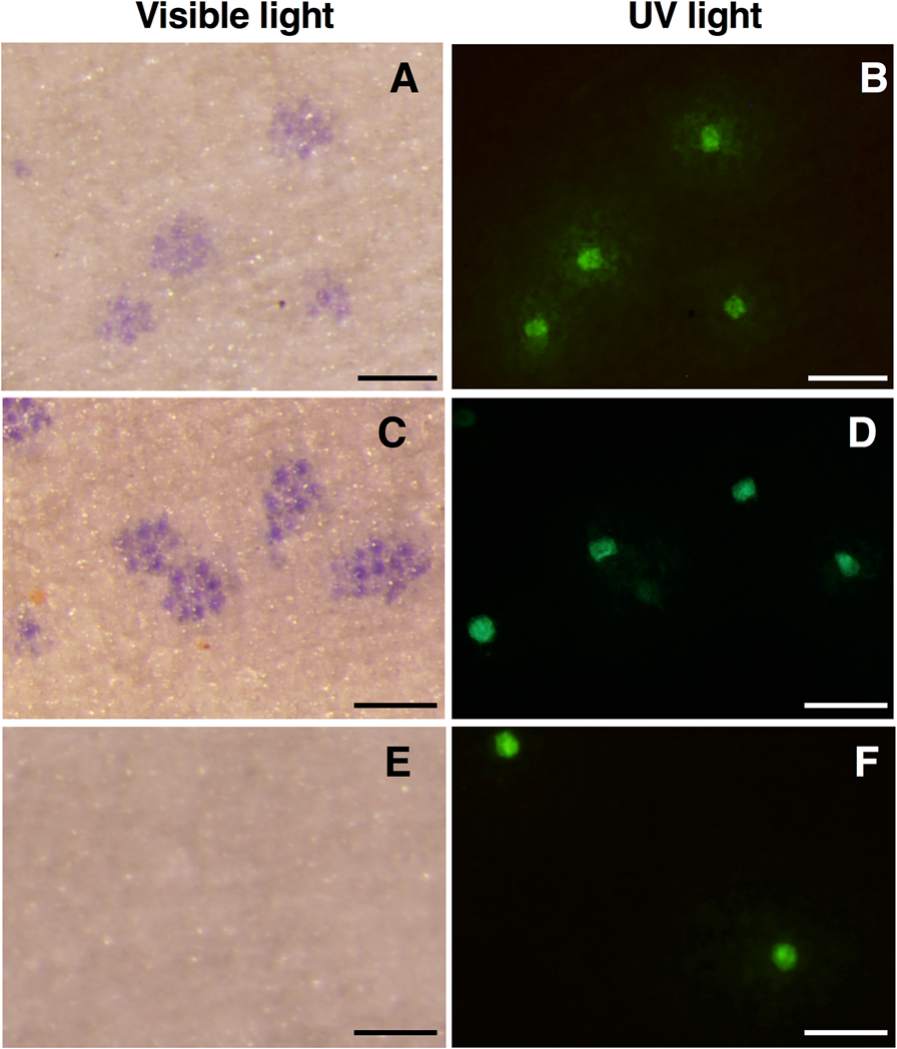
Complementation of the edited GST1 by particle bombardment. Petals of genome-edited line #12 were bombarded with gold particles containing p35S-GST1–1 (**a**, **b**), p35S-GST1–2 (**c**, **d**), and p35S-GUS (**e**, **f**). The petals were also bombarded with particles containing p35S-sGFP, which served as the transformation control. After 24 h, the petals were analyzed microscopically under visible (**a,c**, **e**) and UV light (**b**, **d**, **f**). Bars indicate 100 μm

To confirm whether the white coloration was due to the GST1 deficiency caused by genome editing, we performed a complementation assay involving transient *GST1* expression via particle bombardment. The adaxial side of the petals of *GST1* genome-edited line #12 were bombarded with the p35S-GST1–1 (or p35S-GST1–2) and p35S-sGFP recombinant plasmids just before anthesis. About 24 h after the bombardment, blue cells reflecting the recovery of anthocyanin accumulation were observed against a white background in some epidermal samples examined with a light microscope (Fig. 6a and c). The GFP fluorescence was also observed in the same cells under UV light (Fig. 6b and d). A bombardment with p35S-GUS and p35S-sGFP did not produce blue cells, and only GFP florescence was detected (Fig. 6e and f). These results indicated that the anthocyanin accumulation of *GST1* genome-edited line #12 was restored by the transient expression of *GST1*.

### Induction of anthocyanin pigmentation in leaves

**Fig. 7:**
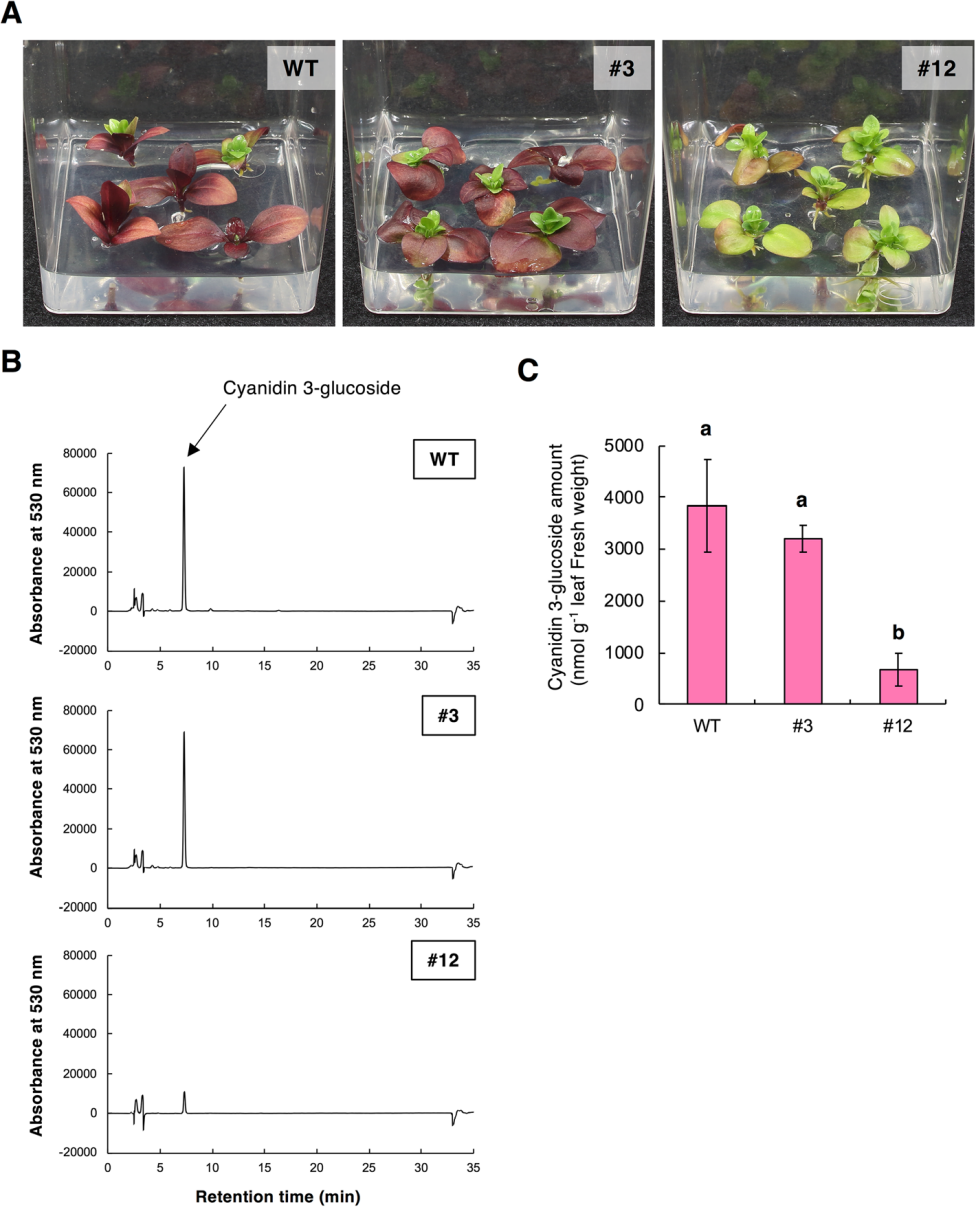
Induction of anthocyanin accumulation in the leaves of *GST1* genome-edited gentian lines. **a** Appearance of WT and *GST1* genome-edited gentian plants on half-strength MS medium supplemented with 10% sucrose at 43 days after being transferred. **b** HPLC chromatograms at 365 nm of leaf extracts from plants grown on half-strength MS medium supplemented with 10% sucrose at 43 days after being transferred. The main peak is cyanidin 3-glucoside. **c** Anthocyanin contents in the leaves of the WT control and *GST1* genome-edited lines. Quantities were determined based on the standard curve generated from the HPLC peak area of six serial dilutions of cyanidin 3-glucoside. Error bars represent the standard error of the means of five leaves from individual plants. Different letters indicate statistically significant differences according to Tukey’s HSD test (*P* < 0.01)

Anthocyanin production is induced by several factors, including low temperatures, high intensity light, osmotic stress, and pathogen infections. To investigate whether *GST1* genome-edited lines can accumulate anthocyanins in response to stress, we cultured them in a sugar-rich medium that usually induces anthocyanin accumulation. The WT gentian plants turned red because of the accumulation of cyanidin 3-glucoside as the major anthocyanin pigment (Fig. 7a and b). However, *GST1* genome-edited line #12 plants did not turn red and relatively little cyanidin 3-glucoside accumulated. The line #3 plants, which produced pale blue flowers, exhibited a red coloration that was similar to that of WT plants, and there were no significant differences in the accumulation of cyanidin 3-glucoside between line #3 and WT plants (Fig. 7c).

## Discussion

Anthocyanins accumulate in various plant organs such as petals, fruits, seed coats, stems, leaves, and roots. These compounds are flavonoids and their contribution to flower colors has been thoroughly reviewed [23]. Additionally, their beneficial effects on human health have also been studied [24]. The flavonoid biosynthesis pathway has been well characterized in higher plants, and includes biosynthesis enzymes (e.g., chalcone synthase to anthocyanin synthase) as well as anthocyanin modification enzymes that catalyze glycosylations and acylations (Fig. 5a). Recent studies revealed that a structural-functional complex called a metabolon is constructed from flavonoid biosynthesis-related enzymes at the endoplasmic reticulum (ER) for the efficient biosynthesis of flavonoid compounds [25]. The transcriptional regulation of these biosynthesis genes via transcription factors, including, MYB, bHLH, and WD40, has been elucidated in many plant species [26]. As mentioned above, the biosynthesis genes and several transcription factor genes involved in gentiodelphin biosynthesis have been characterized in Japanese gentian.

Because of their water solubility, anthocyanins accumulate in vacuoles, which are membrane-bound cellular organelles present in all plants. It has been suggested that the transport of anthocyanins to vacuoles occurs via multidrug resistance-associated ABC transporters [27] in a pathway that requires the conjugation of glutathione (GSH) to anthocyanins by GSTs. However, the mechanisms underlying the involvement of GSTs in anthocyanin transport remain unknown because GSH-conjugated anthocyanins have not been clearly confirmed in most cases. The Phi clade GSTs, such as AN9 (petunia) [28], AcGST1 (kiwifruit) [14], and CsGSTa (tea) [15], reportedly bind to several flavonoids. Thus, these GSTs may function as cytoplasmic flavonoid carrier proteins to transfer anthocyanins from the ER to the tonoplast.

In this study, we selected a candidate Phi-type anthocyanin-related *GST* gene from a draft transcriptome constructed by de novo assembly based on RNA-seq data for *Gentiana triflora* petals and leaves (DRA010021; BioProject PRJDB9616). On the basis of this contig sequence, *GST1* was isolated from the ‘Albireo’ cultivar (*Gentiana triflora* × *Gentiana scabra*) (i.e., WT) and functionally analyzed. Specifically, we targeted *GST1* with the CRISPR/Cas9 genome editing system, which has been used to functionally characterize gentian genes [22]. The biallelic *GST1* mutation efficiency was calculated as 13.0% (7/54 lines) based on the Sanger sequencing and NGS amplicon sequencing data (Additional file 1: Tables S1 and S2). This efficiency was slightly better than those of previous studies regarding the gentian anthocyanin modification genes *Gt5GT* (5.7%), *Gt3′GT* (4.9%), and *Gt5/3′AT* (8.6%) [22]. The diversity in the efficiencies was considered to be dependent on the target sequences. Of the five lines with flower colors that varied from the WT flowers, we focused on two typical *GST1* knockout lines, #3 and #12, based on the extent of the flower color change.

The phenotypic change in line #3 was relatively mild, with flowers that were slightly paler than the blue WT gentian flowers. During the analysis of the NGS amplicon of the target site region in genomic DNA and transcripts, the allele 1 fragment of line #3 was not constructed with a fastq-join script because of the insertion of a specific long sequence derived from the chloroplast genome after a sequence deletion between target sites 1 and 2 (data not shown). Thus, we considered that this mutation eliminated the translation of a functional protein from allele 1. Additionally, we speculated that the deletion in allele 2 of line #3 was not a crucial mutation because it resulted in the deletion of only three amino acids without a frameshift. As indicated in Fig. 3a, we observed a mild flower color change in line #3 and there was no substantial decrease in the anthocyanin content compared with the WT level (Fig. 4b). Furthermore, there were no differences in the hue angles at the petal surface between line #3 and WT plants (Fig. 3b). The peak patterns in the petal extract HPLC chromatograms were also the same between line #3 and WT plants, with the same minor peaks detected (Fig. 4a). These results suggested that the deletion at target site 1 of *GST1* exon 3, which eliminated three amino acids, resulted in a functional protein that was slightly less active than normal. A consequence of this decreased activity was the slight inhibition of gentiodelphin accumulation that caused the mild flower color change (i.e., *GST1* knockdown).

In contrast, in *GST1* genome-edited line #12, the flowers were faint blue to nearly white. Moreover, the total anthocyanin content in line #12 was less than 10% of that of the WT control. The biallelic genome editing was confirmed for both target sites 1 and 2 (Table 1). The mutations were considered to cause frameshifts and unusual stop codons. Other *GST1* genome-edited lines (i.e., #13, #23, and #29), which were also confirmed to have biallelic mutations at the target sites (Additional file 1: Table S1), produced flower colors similar to those of line #12 (Additional file 2: Fig. S1). Thus, we concluded the genome editing of *GST1* was successful, with lines #12, #13, #23, and #29 exhibiting a gene knockout phenotype in contrast to the gene knockdown phenotype of line #3.

Because it was unclear whether the observed phenotypes were due to GST1 deficiency, we performed a complementation assay. The blue coloration of petals was restored in *GST1* genome-edited line #12 by the transient expression of either *GST1–1* or *GST1–2* (Fig. 6), indicating the white-flowered phenotype was indeed caused by a *GST1* mutation. The importance of GST1 was also demonstrated. Specifically, even if the anthocyanin biosynthesis pathway is active, the final transport mediated by GST1 is necessary for the accumulation of anthocyanins in gentian plants.

The qRT-PCR data for developing flowers revealed similar expression patterns among *GST1*, *CHS*, and *CHI*, with early expression that peaked at stage 2. In this stage, *GST1* expression levels were significantly lower in both *GST1* genome-edited lines than in the WT plants. This may have been related to nonsense-mediated mRNA decay (i.e., eukaryotic mRNA quality control) [29, 30]. Transcription factors that regulate the expression of anthocyanin-related *GST* genes have been identified in kiwifruit (AcMYBF110 for *AcGST1*) [14] and apple (MdMYB1 for *MdGSTF6*) [16]. On the basis of the gentian *GST1* expression pattern (Fig. 5b), it is unlikely that GtMYB3 regulates *GST1* expression. The downregulated expression of *GST1* did not significantly affect the expression levels of the examined flavonoid-biosynthetic genes, suggesting feedback regulation via GST did not influence anthocyanin accumulation. Unfortunately, we did not identify the transcription factor regulating *GST1* expression in gentian plants. Therefore, additional studies are necessary to clarify *GST1* transcriptional regulation.

Anthocyanin accumulation is induced by an exogenous sugar treatment, and the regulatory genes involved in the associated sucrose signaling have been characterized [31, 32]. In this study, we observed that the sugar-induced anthocyanin pigmentation in in vitro cultured plants was inhibited in *GST1* genome-edited lines (Fig. 7). This inhibition was severe in knockout line #12, which was consistent with the flower color phenotype. These results implied that *GST1* also contributes to the stress-induced anthocyanin accumulation in gentian plants, although more detailed analyses of *GST* expression in other organs are necessary. From a practical perspective, leaf pigmentation is usually an undesirable trait for ornamental flowers. Therefore, the mechanism regulating stress-induced anthocyanin accumulation via anthocyanin transport should be elucidated.

Our *GST1* genome-edited gentian lines did not produce completely white flowers, implying other proteins are also involved in the anthocyanin transport of gentian plants. We selected and analyzed a Phi (F) class GST in this study. However, several genes encoding Tau (U) class GSTs, such as *Bz2* in *Zea mays* [33] and *VviGST1* in *Vitis vinifera* [34], are also associated with anthocyanins. More recently, *CsGSTb* and *CsGSTc* from *Camellia sinensis* [15] were confirmed as Tau clade *GST* genes related to anthocyanin transport. Our phylogenetic analysis (Fig. 1a) indicated that a *GST* gene, TRINITY_DN42846_c0_g1_i1, which was detected based on the de novo gentian transcripts, belongs in the Tau clade, with deduced amino acid sequence identities of 42, 54, and 55% with VviGST1, CsGSTb, and CsGSTc, respectively. Thus, future studies should examine Tau-type GSTs as well as the ABC transporters to elucidate the mechanism underlying anthocyanin accumulation in gentian plants.

## Conclusions

In this study, we isolated and analyzed an anthocyanin-related *GST* gene, *GST1*, in cultivated Japanese gentian. Its contribution to flower pigmentation was confirmed with the CRISPR/Cas9 genome editing system. We produced several *GST1* genome-edited lines and analyzed the effects of their mutations on flower color. The data presented herein revealed two distinct phenotypes, namely knockout (almost white) and knockdown (pale blue) phenotypes. These results suggest genome editing with the CRISPR/Cas9 system can be applied to knock out target genes as well as slightly modify an allele of a gene of interest. Our study findings may be relevant for clarifying the anthocyanin transport in higher plants. Moreover, our mutant lines may be useful for gentian breeding because such mutations have not been detected in natural mutants. Our results indicate that *GST1* helps mediate the anthocyanin accumulation in gentian plants, although additional related genes remain to be identified. The use of genome editing technology will enhance future studies of anthocyanin transport in gentian plants.

## Methods

### Plant materials and the selection of transformants

Blue-flowered gentian cultivar ‘Albireo’ (*Gentiana triflora* × *Gentiana scabra*) (i.e., WT) was used for the targeted genome editing with the CRISPR/Cas9 system. This cultivar, which was identified by Dr. Masahiro Nishihara, was bred in Iwate prefecture and is registered in the Plant Variety Protection database at Ministry of Agriculture, Forestry and Fisheries, Japan (registration no. 2553). It was kindly provided by the Iwate Agricultural Research Center. The WT plants were maintained in an in vitro culture using half-strength MS medium supplemented with 3% sucrose as the basal medium under the same cultivation conditions in our previous study [22].

Gentian plants were transformed according to an *A. tumefaciens*-mediated procedure, after which the genome-edited transformants were selected as previously described [22, 35]. Briefly, transformed shoots were screened on medium containing 0.75 mg l^− 1^ bialaphos. Candidate transgenic plants were selected based on the PCR amplification of a pcoCas9 fragment with MightyAmp DNA polymerase (TaKaRa, Shiga, Japan). Primers #61 and #62, which are specific for pcoCas9, were used for the PCR analysis of crude leaf extracts as the template according to the manufacturer’s instructions. The genome-edited plants were selected by detecting insertions or deletions in the target sites via direct- and subcloning-sequence analyses of the PCR products amplified with primers #154 and #155 specific for the target site region and the above-described method (Fig. 2a). Details regarding all primers are listed in Additional file 1: Table S3. The regenerated *GST1* genome-edited lines were acclimated to the closed greenhouse or cultured in an incubation room under light-emitting diode lamps as described previously until they bloomed [36]. Petals and leaves were harvested, immediately frozen in liquid nitrogen, and stored at − 80 °C until analyzed.

### Isolation of *GST* cDNA and genomic sequences related to anthocyanin accumulation

Single strand cDNAs were synthesized from the total RNA extracted from the petals of WT plants and used for PCR amplification as described previously [22]. The sequences used for designing primers specific for gentian *GST* genes were obtained from the DDBJ DRA database (accession no. DRA010021). From more than 130,000 contigs, 30 contigs were identified as *GST* genes based on BLASTX (version 2.2.26) matches, including one that was annotated with the GO term “anthocyanin-containing compound metabolic process”. We designed primers #276 and #277 based on the sequencing data for this *GST* contig for the subsequent 3′-rapid amplification of cDNA ends with the GeneRacer kit (Invitrogen, USA). The full-length *GST1* sequence was amplified by PCR with primers #325 and #326, after which the amplicon was sequenced with the Big-Dye Terminator Cycle Sequencing kit (version 1.1) and the ABI PRISM 3130xl or 3500 Genetic Analyzers (Applied Biosystems, Foster City, CA, USA).

To isolate the full-length *GST1* genomic sequence, genomic DNA was isolated from the young leaves of WT gentian plants with the GeneElute Genomic DNA Isolation system (Sigma-Aldrich, St Louis, MO, USA). The PCR amplification with primers #325 and #326 and the sequencing of the amplicon were performed as described above. The primers used for isolating *GST1* are listed in Additional file 1: Table S3. The *GST* coding regions were translated to the respective deduced amino acid sequences with ExPASy (https://web.expasy.org/translate/). A phylogenetic tree was produced and GST amino acid sequences were aligned with the CLUSTALW (Thompson et al. 1994), MEGA (version 10.1.7), and Jalview (version 2.10.3) programs. The GST protein sequences used for constructing the phylogenetic tree are listed in Additional file 1: Table S4.

### Construction of a binary vector for editing *GST1* in the gentian genome

A binary CRISPR/Cas9 vector for targeting gentian *GST1* was constructed to harbor two single-guide RNA expression cassettes using the same procedure in our previous study [22]. The two target sites in *GST1* exon 3 are presented in Fig. 2a. The resultant binary vector pSbar-pcoCas9-AtU6-26p-GST1target-1gRNA-AtU6-26p-GST1target2gRNA (Fig. 2b) was transformed into *A. tumefaciens* strain EHA101 by electroporation and used for gentian transformation.

### Next-generation sequencing analysis of the genome-edited target site region of *GST1*

The genomic DNA and transcripts in the petals of the *GST1* genome-edited lines and WT plants were analyzed with the Illumina MiSeq Next-generation Sequencer to confirm the target site sequences. Genomic DNAs and cDNAs were prepared as described above. The extracted RNA samples were reverse transcribed with PrimeScript II (TaKaRa). Amplicon sequencing was performed on an Illumina system as described previously [21]. Briefly, raw reads were pre-processed with the FASTX toolkit, after which a single contiguous sequence (fragment) containing the target site region was constructed with a fastq-join script [37]. Finally, all unique fragments were counted. The primers used for the NGS analysis are listed in Additional file 1: Table S1.

### Measurement of gentian petal colors

Color differences were determined based on CIE L*a*b*, in which L* indicates lightness, a* is the red/green coordinate, and b* is the yellow/blue coordinate. To quantify the flower color phenotypes of the *GST1* genome-edited lines and WT plants, the colorimetric values L*, a*, and b* as well as the chroma (C*) value and hue angle, which were calculated based on the L*, a*, and b* values, of the adaxial surface of the limb area of fresh petals were measured. The benchtop CM-3600A spectrophotometer (Konica Minolta, Tokyo, Japan) was used for this analysis.

### Analysis of anthocyanin compositions in the petals of *GST1* genome-edited gentian plants

The anthocyanin compositions of petal extracts were analyzed by HPLC system [PU-4180 PUMP, MD-4010 photodiode array detector, and ChromNAV (version 2.03.05) software; JASCO, Tokyo, Japan] equipped with a Unifinepak C18 column (4.6 mm internal diameter × 250 mm; JASCO). Anthocyanins were extracted from fresh petals and subjected to HPLC analysis as described previously [22]. We used a linear gradient elution (1.1 ml min − 1) of 14–22% acetonitrile in 1% aqueous phosphoric acid over 30 min. The V-730BIO spectrophotometer (JASCO) was used to estimate anthocyanin concentrations based on the molar absorptivity of delphinidin chloride [ε mol = 506,783 at 530 nm, evaluated in the 80% methanol solution containing 0.1% (v/v) trifluoroacetic acid].

### Expression analysis of *GST1* and flavonoid biosynthesis-related genes

Total RNA was extracted from flower petals collected at four developmental stages as described above, after which cDNA was synthesized with the PrimeScript™ RT reagent Kit with gDNA Eraser (Perfect Real Time) (TaKaRa) according to the manufacturer’s instructions. A qRT-PCR assay was completed with TB Green™ Premix Ex Taq™ II (Tli RNaseH Plus) (TaKaRa) and the QuantStudio 5 Real-Time PCR system (Applied Biosystems Japan). The reaction mixtures (20 μl total volume) consisted of the following components: 10 μl master mix, 0.8 μM each primer, and 1 μl cDNA template. The qRT-PCR primer sets for *GST1*, *CHS*, *CHI*, *F3′5′H*, *DFR*, *ANS*, *5/3′AT*, and *MYB* are listed in Additional file 1: Table S3. The PCR conditions were as follows: 50 °C for 2 min; 95 °C for 10 min; 40 cycles of 95 °C for 15 s and 60 °C for 1 min. A melting curve analysis was performed to verify the specificity and identity of the qRT-PCR products. The expression levels of each gene were normalized against the *UBQ* expression level and recorded as relative values based on the maximum expression levels.

### Complementation of the edited *GST1* by particle bombardment

In this study, p35S-sGFP, containing *sGFP* under the control of the cauliflower mosaic virus 35S promoter, was used as the transformation control plasmid, whereas p35S-GUS was used as the negative control plasmid. The *sGFP* fragment was replaced with a modified *GST1–1* (or *GST1–2*) (six base substitutions in the target site; details in Additional file 2: Fig. S2) designed to disrupt the editing by the integrated CRISPR/Cas9 construct, resulting in p35S-GST1–1 and p35S-GST1–2. The recombinant plasmids were co-precipitated onto gold particles. The PDS-1000/He Biolistic Particle Delivery System (Bio-Rad, CA) was used to bombard the *GST1* genome-edited gentian petals with the particles as previously described [38]. After 24 h, the petals were analyzed microscopically under visible and UV light.

### Induction of anthocyanin pigmentation in leaves

The WT and *GST1* genome-edited gentian plants maintained by in vitro cultivation were transferred to half-strength MS medium supplemented with 10% sucrose. Plants were grown in a culture room set at 22 °C under a 16-h light/8-h dark photoperiod, with light supplied by white-light-emitting diodes. An HPLC analysis was performed as described above. Anthocyanin contents were estimated based on the standard curve generated from the HPLC peak area of six serial dilutions of cyanidin 3-glucoside.

## Supplementary information

**Additional file 1: Table S1.** Sanger sequencing analysis of the PCR amplicons of the DNA from *GST1* genome-edited lines and the WT control. **Table S2.** Next-generation sequencing analysis of the PCR amplicons of the DNA and RNA from *GST1* genome-edited lines and the WT control. **Table S3.** Primers used in this study. **Table S4.** Glutathione *S*-transferase proteins used for constructing the phylogenetic tree in Fig. 1a.

**Additional file 2: Figure S1.** Flower color characteristics in *GST1* genome-edited gentian lines #13, #23, and #29. (A) Flower color phenotypes of *GST1* genome-edited lines #13, #23, and #29. (B) L*, a*, and b* color values at the surface of fresh petals were measured with the CM-3600A spectrophotometer (Konica Minolta, Tokyo, Japan). The chroma values and hue angles were also calculated. **Figure S2** Sequence of the third exon of the modified *GST1* for the complementation assay. The highlighted nucleotides (i.e., shaded, bold, or boxed) are the same as those in Fig. 2b. The substituted nucleotides for the transient expression assay are indicated in red. These substitutions did not change the encoded amino acids.

**Additional file 3 **The results of next-generation amplicon sequencing of WT and *GST1* genome-edited gentian lines. Numbers after sequence names in the Excel worksheets are sequential serial numbers and read counts of each fragment.

## Data Availability

The data generated during this study are included in this published article and its additional files. Primary datasets used and/or analyzed during the current study available from the corresponding author on reasonable request.

## Abbreviations

GST: Glutathione *S*-transferase
CRISPR: Clustered regularly interspaced short palindromic repeats
NGS: Next-generation sequencing
PCR: Polymerase chain reaction
HPLC: High-performance liquid chromatography
