# Supplementary material for: Molecular characterization of an anthocyanin-related glutathione S-transferase gene in Japanese gentian with the CRISPR/Cas9 system
Source: BMC Plant Biol. 2020 Aug 6;20:370. doi: 10.1186/s12870-020-02565-3 (PMC7409652; doi:10.1186/s12870-020-02565-3)
Supplement: Supplementary file 1 — Additional file 1: Table S1. Sanger sequencing analysis of the PCR amplicons of the DNA from GST1 genome-edited lines and the WT control. Table S2. Next-generation sequencing analysis of the PCR amplicons of the DNA and RNA from GST1 genome-edited lines and the WT control. Table S3. Primers used in this study. Table S4. Glutathione S-transferase proteins used for constructing the phylogenetic tree in Fig. 1a. [file 12870_2020_2565_MOESM1_ESM.pdf]

Supplementary Table S1. Sanger sequencing analysis of the PCR amplicons of the DNA from *GST1* genome-edited lines and the WT control

<table>
<tr><th rowspan="2">Line no.</th><th rowspan="2">Allele</th><th colspan="3">Number of sequenced clones</th><th rowspan="2">Target1</th><th rowspan="2">In/Del</th><th rowspan="2">Target2</th><th rowspan="2">In/Del</th></tr>
<tr><th>Total</th><th colspan="2">Number</th></tr>
<tr><td>WT</td><td>1, 2</td><td></td><td colspan="2"></td><td>GGGTATACCCACCGATTCTTCGG</td><td></td><td>GCTGATATGTGCCACCTGCCGGG</td><td></td></tr>
<tr><td rowspan="2">#1</td><td>1</td><td rowspan="2">3</td><td>2</td><td>2</td><td>GGGTATACC-----||------</td><td>-24</td><td>GCTGATATGTGCCACCTGCCGGG</td><td>0</td></tr>
<tr><td>2</td><td>1</td><td>1</td><td>GGGTATACCCACCGATTCTTCGG</td><td>0</td><td>GCTGATATGTGCCACCTGCCGGGG</td><td>+1</td></tr>
<tr><td rowspan="3">#3</td><td>1</td><td rowspan="3">5</td><td>0</td><td>0</td><td colspan="4">No detection</td></tr>
<tr><td rowspan="2">2</td><td rowspan="2">5</td><td>4</td><td>GGGTATAC---------CTTCGG</td><td>-9</td><td>GCTGATATGTGCCACCTGCCGGG</td><td>0</td></tr>
<tr><td>1</td><td>GGGTATAC---------CTTCGG</td><td>-9</td><td>GCTGATATGTGCCACCTGCCGGGG</td><td>+1</td></tr>
<tr><td rowspan="3">#8</td><td>1</td><td rowspan="3">5</td><td>9</td><td>9</td><td>GGGTATACC------||-----</td><td>-15</td><td>GCTGATATGTGCCAC--GCCGGG</td><td>-2</td></tr>
<tr><td rowspan="2">2</td><td rowspan="2">2</td><td>1</td><td>GGGTATACCCACCGATTCTTCGG</td><td>0</td><td>GCTGATATGTGC-----GCCGGGG</td><td>-5, +1</td></tr>
<tr><td>1</td><td>GGGTATACCCACCGATTCTTCGG</td><td>0</td><td>GCTGATATGTGC-----GCCGGGGG</td><td>-5, +2</td></tr>
<tr><td rowspan="4">#12</td><td rowspan="3">1</td><td rowspan="4">7</td><td rowspan="3">5</td><td>1</td><td>--------||-------TTCGG</td><td>-19</td><td>GCTGATAT-------CTGCCGGG</td><td>-7</td></tr>
<tr><td>1</td><td>--------||-------TTCGG</td><td>-19</td><td>GCTGATATGTGCC-------GGGGGG</td><td>-7,+3</td></tr>
<tr><td>3</td><td>--------||-------TTCGG</td><td>-19</td><td>GCTGATATGT-------GCCGGG</td><td>-7</td></tr>
<tr><td>2</td><td>2</td><td>2</td><td>GGTATACCCACCGA--CTTCGG</td><td>-2</td><td>GCTGATATGTGCCAC--GCCGGG</td><td>-2</td></tr>
<tr><td rowspan="2">#13</td><td>1</td><td rowspan="2">7</td><td>7</td><td>7</td><td>GGGTATACCCACCGA-TCTTCGG</td><td>-1</td><td>GCTGATATGTGC-----GCCGGG</td><td>-5</td></tr>
<tr><td>2</td><td>0</td><td>0</td><td></td><td></td><td></td><td></td></tr>
<tr><td rowspan="2">#23</td><td>1</td><td rowspan="2">5</td><td>5</td><td>5</td><td>GGGTATACCCACCGATTCTTCGG</td><td>0</td><td>GCTGATATGTGCCAC--GCCGGG</td><td>-2</td></tr>
<tr><td>2</td><td>0</td><td>0</td><td></td><td></td><td></td><td></td></tr>
<tr><td rowspan="2">#29</td><td>1</td><td rowspan="2">9</td><td>0</td><td>0</td><td colspan="4">No detection</td></tr>
<tr><td>2</td><td>9</td><td>9</td><td>GGGTATACCCA------CTTCGG</td><td>-6</td><td>GCTGATATGTGC-----GCCGGG</td><td>-5</td></tr>
</table>

Supplementary Table S2. Next-generation sequencing analysis of the PCR amplicons of the DNA and RNA from *GST1* genome-edited lines and the WT control

Line no.	Allele	Number of fragments (DNA)	Number of fragments (RNA)	Target1	In/Del	Target2	In/Del	Flower color phenotype
WT	1	9,782	20,438	GGGTATACCCACCGATTCTTCGG	0	GCTGATATGTGCCACCTGCCGGG	0	Blue
	2	10,199	3,992	GGGTATACCCACCGATTCTTCGG	0	GCTGATATGTGCCACCTGCCGGG	0	
#3	1	-	-	Large insertion			-	Bale blue
	2	25,763	31,764	GGGTATAC---------CTTCGG	-9	GCTGATATGTGCCACCTGCCGGG	0	
#12	1	27,408	17,831	-------------------TCGG	-19	GCTGATATGT-------GCCGGG	-7	Almost white color
	2	26,029	3,222	GGGTATACCCACCGA--CTTCGG	-2	GCTGATATGTGCCAC--GCCGGG	-2	
#13	1	12,587	18,989	GGGTATACCCACCGA-TCTTCGG	-1	GCTGATATGTGC-----GCCGGG	-5	Almost white color
	2	13,805	1,728	GGGTATACCCAC-----CTTCGG	-5	GCTGATATGTGCCACCTGCCGGG	0	
#23	1	19,080	29,053	GGGTATACCCACCGATTCTTCGG	0	GCTGATATGTGCCAC--GCCGGG	-2	Almost white color
	2	27,740	12,542	GGGTATACC-------------------||--------------------GCCGGG			-129	
#29	1	-	-	Large insertion			-	Almost white color
	2	24,369	18,889	GGGTATACCCA------CTTCGG	-6	GCTGATATGTGC-----GCCGGG	-5	

Supplementary Table S3. Primers used in this study

#	Primer name	Sequence (5'-3')	Index
	**Isolation of GST1**		
	**3' RACE PCR**		
#276	GST1-1_3race_first	GTCGATCAATGGCTGGAAGT	
#277	GST1-1_3race_nested	AACAAGCTCCAAAACGTGCT	
	**Full-length amplification**		
#325	Alb.GST1_5'UTR_F1	TAAAGGTTTAGCAGAAGAAAAATAGCA	
#326	Alb.GST1_3'UTR_R1	CCGTATTAAACATTTCTACTACCACT	
	**Genome sequence for 2_{nd} intron**		
#98	GST1_exon2_Fw	TTCCTGTCATTGAAGATGGTGA	
#99	GST1_exon3_Rv	AAGTGGTTCCGAGGAGGTTT	
	**Direct sequence**		
#154	GST1_U238	GACAAGGGACCAAACCTCCT	
#155	GST1_L639	TTTCATCAAATCGAGCACTTTC	
	**Selection of genome-edited transformants**		
#61	Cas9U1898	AAGAAAGGCTCAAGACCTACGCTCAT	
#62	Cas9L2260	TCGATCACGATGTTCTCAGGCTTATG	
	**Amplicon sequence**		
	**1st PCR primer sets**		
#404	Alb.GST1tar1,2_D50X-2	ACACTCTTTCCCTACACGACGCTCTTCCGATCtGACAAGGGACCAAACCTCCT	
#365	Alb.GST2tar1,2_D70X	TGACTGGAGTTCAGACGTGTGCTCTTCCGATCtCCTTAATCAAATCCCCAACG	
	**2nd PCR forward primers**		
	**D503**	AATGATACGGCGACCACCGAGATCTACACCCTATCCTACACTCTTTCCCTACACGACG	CCTATCCT
	**D504**	AATGATACGGCGACCACCGAGATCTACACGGCTCTGAACACTCTTTCCCTACACGACG	GGCTCTGA
	**D505**	AATGATACGGCGACCACCGAGATCTACACAGGCGAAGACACTCTTTCCCTACACGACG	AGGCGAAG
	**D506**	AATGATACGGCGACCACCGAGATCTACACTAATCTTAACACTCTTTCCCTACACGACG	TAATCTTA
	**D507**	AATGATACGGCGACCACCGAGATCTACACCAGGACGTACACTCTTTCCCTACACGACG	CAGGACGT
	**D508**	AATGATACGGCGACCACCGAGATCTACACGTACTGACACACTCTTTCCCTACACGACG	GTACTGAC
	**2nd PCR reverse primers**		
	**D710**	CAAGCAGAAGACGGCATACGAGATTTCGCGGAGTGACTGGAGTTCAGACGTGT	TCCGCGAA
	**D711**	CAAGCAGAAGACGGCATACGAGATGCGCGAGAGTGACTGGAGTTCAGACGTGT	TCTCGCGC
	**D712**	CAAGCAGAAGACGGCATACGAGATCTATCGCTGTGACTGGAGTTCAGACGTGT	AGCGATAG
	**Real Time PCR**		
#5	GST1-RT-F	GCGTTGGGGATTTGATTAAG	
#6	GST1-RT-R	TCGAGCACTTTCTTCCAAGC	
#19	CHS-RT-F	ATGGTTGAAGTCCCAAAACTTG	
#20	CHS-RT-R	CCCCATTCTTTAATTGCCTTCT	
#7	CHI-RT-F	TGGAGCTATTCCTGAAGTAAGCA	
#8	CHI-RT-R	TGCAGCTGGTGAAACACCTT	
#21	F3′5′H-RT-F	GCTTAAAGCCATGTTTGAATCG	
#22	F3′5′H-RT-R	CGTTAGCATCTCCGAAATCATC	
#13	DFR-RT-F	CACGAATTTGACGTTGTTGAAG	
#14	DFR-RT-R	AAACACCATGACAGCCATGAAT	
#15	ANS-RT-F	ACCCAGATAGTACCAACCATCG	
#16	ANS-RT-R	GTTCTTTAATTCGCCGTGACAT	
#17	5/3′AT-RT-F	GCAGTCCCTTCTGTTTTACGAC	
#18	5/3′AT-RT-R	AGAGTGAGAGACAAAGAGGCCTTA	
#11	MYB3-RT-F	CACCGGATGAAGAAGATTTGAT	
#12	MYB3-RT-R	ATCAGTCCTTCCTGGGATTCTC	
#9	UBQ-RT-F	GGGAAGCAGTTGGAAGATGG	
#10	UBQ-RT-R	TTCAGAAACCACCACGGAGA	

Supplementary Table S4. Glutathione *S*-transferase proteins used for constructing the phylogenetic tree in Figure 1A

Gene name	GST class	Database	Accession Number	Plant species
BZ2	*Tau*	NCBI	NP_001183661.1	*Zea may*
GmHSP26-A	*Tau*	NCBI	NP_001238439.1	*Glycine max*
VvGST1	*Tau*	NCBI	NP_001268177.1	*Vitis vinifera*
CsGSTb	*Tau*	GeneBank	MK431868	*Camellia sinensis*
CsGSTc	*Tau*	GeneBank	MK431869	*Camellia sinensis*
AtGSTU1	*Tau*	NCBI	XP_020885836.1	*Arabidopsis thaliana*
AtGSTU10	*Tau*	NCBI	NP_177598.1	*Arabidopsis thaliana*
AtGSTU11	*Tau*	NCBI	NP_177151.1	*Arabidopsis thaliana*
AtGSTU17	*Tau*	GeneBank	OAP15816.1	*Arabidopsis thaliana*
AtGSTL1	*Lambda*	NCBI	NP_001119157.1	*Arabidopsis thaliana*
AtGSTL2	*Lambda*	NCBI	NP_191064.1	*Arabidopsis thaliana*
AtGSTL3	*Lambda*	NCBI	NP_195899.1	*Arabidopsis thaliana*
GmGSTL3	*Lambda*	NCBI	NP_001304515	*Glycine max*
GST1	*Phi*	This study	LC536038	*Gentiana triflora* x *G. scabra*
AN9	*Phi*	GeneBank	CAA68993.1	*Petunia* x hybrida
RAP	*Phi*	NCBI	XP_004288578	*Fragaria vesca* subsp. *vesca*
VvGST4	*Phi*	NCBI	NP_001267869.1	*Vitis vinifera*
DcGSTF2	*Phi*	GeneBank	BAM21533.1	*Dianthus caryophyllus*
CkmGST3	*Phi*	GeneBank	AB682678.1	*Cyclamen persicum* x *C. purpurascens*
MdGSTF6	*Phi*	NCBI	NP_001315851.1	*Malus domestica*
DcGSTF1	*Phi*	GeneBank	BAM21532.1	*Dianthus caryophyllus*
AcGST1	*Phi*	GeneBank	MK238757.1	*Actinidia chinensis*
CsGSTa	*Phi*	GeneBank	QCQ77495.1	*Camellia sinensis*
AtGSTF2	*Phi*	NCBI	NP_192161.1	*Arabidopsis thaliana*
AtGSTF3	*Phi*	NCBI	NP_178394.1	*Arabidopsis thaliana*
AtGSTF4	*Phi*	NCBI	NP_849581.1	*Arabidopsis thaliana*
AtGSTF5	*Phi*	NCBI	NP_001322019.1	*Arabidopsis thaliana*
AtGSTF6	*Phi*	NCBI	NP_171792.1	*Arabidopsis thaliana*
AtGSTF7	*Phi*	NCBI	NP_171791.1	*Arabidopsis thaliana*
AtGSTF8	*Phi*	NCBI	NP_850479.1	*Arabidopsis thaliana*
AtGSTF9	*Phi*	NCBI	NP_180643.1	*Arabidopsis thaliana*
AtGSTF10	*Phi*	NCBI	NP_180644.1	*Arabidopsis thaliana*
AtGSTF11	*Phi*	NCBI	NP_186969.1	*Arabidopsis thaliana*
AtGSTF12 / TT19	*Phi*	NCBI	NP_197224.1	*Arabidopsis thaliana*
AtGSTF13	*Phi*	NCBI	NP_191835.1	*Arabidopsis thaliana*
AtGSTF14	*Phi*	NCBI	NP_175408.1	*Arabidopsis thaliana*
AtGSTT1	*Theta*	NCBI	NP_198937.1	*Arabidopsis thaliana*
AtGSTT2	*Theta*	NCBI	NP_198940.3	*Arabidopsis thaliana*
AtGSTT3	*Theta*	NCBI	NP_198938.1	*Arabidopsis thaliana*
HsGSTZ1	*Zeta*	NCBI	AAC33591.1	*Homo sapiens*
