# Supplementary material for: Molecular characterization of an anthocyanin-related glutathione S-transferase gene in Japanese gentian with the CRISPR/Cas9 system
Source: BMC Plant Biol. 2020 Aug 6;20:370. doi: 10.1186/s12870-020-02565-3 (PMC7409652; doi:10.1186/s12870-020-02565-3)
Supplement: Supplementary file 2 — Additional file 2: Figure S1. Flower color characteristics in GST1 genome-edited gentian lines #13, #23, and #29. (A) Flower color phenotypes of GST1 genome-edited lines #13, #23, and #29. (B) L*, a*, and b* color values at the surface of fresh petals were measured with the CM-3600A spectrophotometer (Konica Minolta, Tokyo, Japan). The chroma values and hue angles were also calculated. Figure S2 Sequence of the third exon of the modified GST1 for the complementation assay. The highlighted nucleotides (i.e., shaded, bold, or boxed) are the same as those in Fig. 2b. The substituted nucleotides for the transient expression assay are indicated in red. These substitutions did not change the encoded amino acids. [file 12870_2020_2565_MOESM2_ESM.pdf]

A

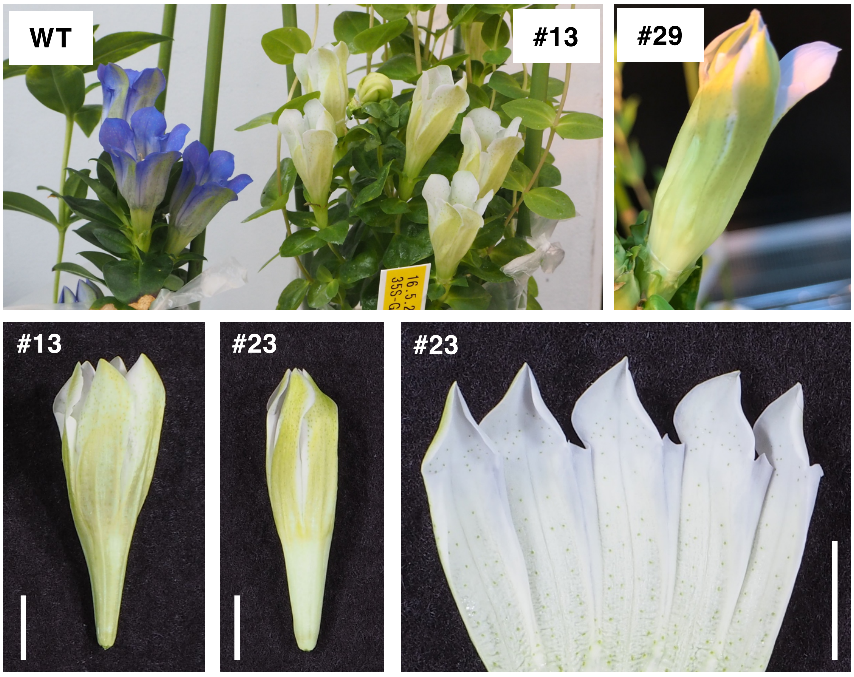

B

Line no.	No. of samples	*L**	*a**	*b**	Chroma	Hue (*h*°)
**WT**	**4**	40.15 ^{a*}	15.84 ^{a}	-49.09 ^{a}	51.59 ^{a}	287.86 ^{a}
**#3**	**5**	47.14 ^{b}	9.10 ^{b}	-40.84 ^{b}	41.86 ^{b}	282.55 ^{a}
**#12**	**7**	82.07 ^{c}	-4.72 ^{c}	1.41 ^{c}	4.97 ^{c}	163.50 ^{b}
**#13**	**4**	82.20 ^{c}	-5.58 ^{c}	7.26 ^{d}	9.16 ^{d}	127.54 ^{c}
**#23**	**4**	77.26 ^{d}	-4.37 ^{c}	-1.50 ^{e}	4.98 ^{c}	197.14 ^{d}
**#29**	**4**	80.22 ^{cd}	-4.14 ^{c}	-2.49 ^{e}	4.85 ^{c}	210.98 ^{d}

* Different letters indicate significant differences at $P < 0.01$ by Tukey's HSD test.

Figure S1

5′-AATCAAGAGCAATAGTAAGATACTATGCAGCAAAGTACGCCGACAAGGGACCAAACCTCCTCGGAA

CCACTTTAGAGGAGAAAGCCGCCGTCGATCAATGGCTGGAAGTTGAGTCCCACAACTTCAACGACT

C A T

TGGTTGTTGCATTTGTCCTTCAAATTGAGGTTCTTCCACGTAT**GGGTATACCCACCGATTCTTCGG**

PAM

TGGTTCAAGACACCATCAACAAGCTCCAAAACGTGCTGGATGTATACGAGCAGAGGCTGTCCCGGA

TT A

GCAAGTATCTCGCCGGAGATGAATTCACCATT**GCTGATATGTGCCACCTGCCGGG**GATTAAGTTTC

PAM

TGACGACTGACGGCGGCGTTGGGGATTTGATTAAGGAGAGGAAGAGTGTGAATTCTTGGTGGGCTG

ATATTTCGAGCCGCCCGGCTTGGAAGAAAGTGCTCGATTTGATGAAAAAA -3′

Figure S2
